# Three New Myrsinol Diterpenes from *Euphorbia prolifera* and Their Neuroprotective Activities

**DOI:** 10.3390/molecules17089520

**Published:** 2012-08-09

**Authors:** Jing Xu, Daqing Jin, Ping Guo, Chunfeng Xie, Lingzhi Fang, Yuanqiang Guo

**Affiliations:** 1 State Key Laboratory of Medicinal Chemical Biology and Tianjin Key Laboratory of Molecular Drug Research, College of Pharmacy, Nankai University, Tianjin 300071, China; 2 School of Medicine, Nankai University, Tianjin 300071, China; 3 Department of Analytical Chemistry, College of Pharmacy, Shenyang Pharmaceutical University, Shenyang 110016, China; 4 State Key Laboratory of Bioactive Substance and Function of Natural Medicines, Institute of Materia Medica, Chinese Academy of Medical Sciences and Peking Union Medical College, Beijing 100050, China

**Keywords:** *Euphorbia prolifera*, diterpenoids, myrsinolditerpenes, neuroprotective activities

## Abstract

Three new myrsinol diterpenes were isolated from the roots of *Euphorbia prolifera*. Their structures were elucidated as 2*α*-*O*-isobutyryl-3*β*,5*α*,7*β*,10,15*β*-penta-*O*-acetyl-14*α*-*O*-benzoyl-10,18-dihydromyrsinol (**1**), 2*α*-*O*-isobutyryl-3*β*-*O*-propion-yl-5*α*,7*β*,10,15*β*-tetra-*O*-acetyl-10,18-dihydromyrsinol (**2**), and 2*α*,14*α*-di-*O*-benzoyl-3*β*,5*α*,7*β*,10,15*β*-penta-*O*-acetyl-10,18-dihydromyrsinol (**3**) on the basis of spectroscopic data analyses (IR, ESI-MS, HR-ESI-MS, and 1D and 2D NMR). Their neuroprotective activities were evaluated and compounds **1** and **2** showed neuroprotective effects against MPP^+^-induced neuronal cell death in SH-SY5Y cells.

## 1. Introduction

**Figure 1:**
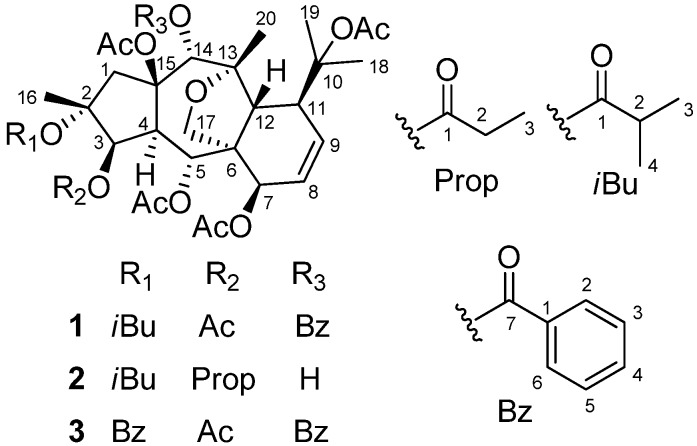
Structures of compounds **1**–**3** from *E. prolifera.*

*Euphorbia prolifera* Buch-Ham, belonging to the family Euphorbiaceae, is a perennial herbaceous plant distributed in southwest China [1]. Its roots have been used as a folk medicine to treat inflammations and tumors [2]. Previous phytochemical investigations on *E. prolifera *revealed that the main and characteristic constituents are myrsinol-type diterpenes [3,4,5,6,7,8,9], which are rare in natural products and whose studies of biological activities are limited [6]. In the course of our survey on new and pharmacologically active substances in medicinal plants [10,11], considerable attention has been given to the occurrence of compounds with neuroprotective effects, since these substances are expected to be useful for the treatment of nervous system diseases, such as Parkinson’s disease [12]. As a continuation of the search for bioactive natural products, our further chemical investigation on the roots of *E. prolifera* led to the isolation of three new myrsinol diterpenes **1-3** (Figure 1). Their structures were elucidated as 2*α*-*O*-isobutyryl-3*β*,5*α*,7*β*,10,15*β*-penta-*O*-acetyl-14*α*-*O*-benzoyl-10,18-dihydromyrsinol (**1**), 2*α*-*O*-isobutyryl-3*β*-*O*-propionyl-5*α*,7*β*,10,15*β*-tetra-*O*-acetyl-10,18-dihydro-myrsinol (**2**), and 2*α*,14*α*-di-*O*-benzoyl-3*β*,5*α*,7*β*,10,15*β*-penta-*O*-acetyl-10,18-dihydromyrsinol (**3**) on the basis of spectroscopic data analyses (IR, ESI-MS, HR-ESI-MS, and 1D and 2D NMR). The neuroprotective activities against 1-methyl-4-phenyl-1,2,3,6-tetrahydropyridine (MPP^+^)-induced neuronal cell death in SH-SY5Y cells were also evaluated. Herein, we describe the isolation and structural elucidation of the three new natural myrsinol diterpenes and their neuroprotective activities.

## 2. Results and Discussion

**Figure 2:**
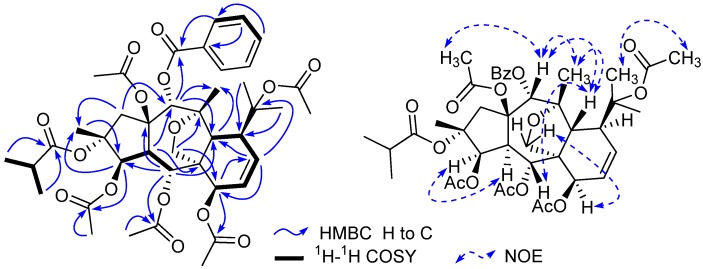
Selected HMBC, ^1^H-^1^H COSY, and NOESY correlations of compound **1**.

Compound **1** was isolated as colorless needles. Its molecular formula was determined as C_41_H_52_O_15_ on the basis of HR-ESI-MS (*m*/*z* 807.3195 [M + Na]^+^, calcd. for C_41_H_52_O_15_Na 807.3204). The ^1^H-NMR spectrum of **1** exhibited two olefinic protons [*δ*_H_ 6.17 (1H, dd, *J* = 10.2, 6.5 Hz, H-8), and 5.90 (1H, dd, *J* = 10.2, 5.6 Hz, H-9)], four oxygenated methines [*δ*_H_ 5.48 (1H, d, *J *= 3.9 Hz, H-3), 5.92 (1H, d, *J *= 11.0 Hz, H-5), 4.84 (1H, d, *J *= 6.5 Hz, H-7), and 5.78 (1H, s, H-14)], and one oxygenated methylene [*δ*_H_ 4.09 and 3.49 (each 1H, d, *J* = 8.8 Hz, H_2_-17)]. In addition, 11 methyls and one monosubstituted benzene ring were also revealed in the ^1^H-NMR spectrum. The ^13^C-NMR spectrum of **1** exihibited 41 carbon resonances. From the ^1^H- and ^13^C-NMR spectra of **1**, five acetyl groups [*δ*_H_ 2.13 s, 2.09 s, 2.05 s, 1.98 s, and 1.96 s; *δ*_C_ 170.6, 170.3, 170.2, 169.2, and 168.4 (CO), and 22.4, 22.2, 21.0, 20.9, and 20.8 (CH_3_)], and one benzoyl group (*δ*_H_ 8.09 × 2 d, 7.42 × 2 t, and 7.56 t; *δ*_C_ 129.6, 129.9 × 2, 128.3 × 2, 133.2, and 165.6) (Table 1 and Table 2) were confirmative, and one isobutyryl group (*δ*_H_ 2.24 q, 0.83 d, and 1.08 d; *δ*_C_ 18.2, 18.8, 34.3, and 175.3) (Table 1 and Table 2) was also deduced based on those reported diterpenes with isobutyryl groups from the genus *Euphorbia* [4,5,7]. Apart from the above 21 signals for the substituents (five acetoxy groups, one isobutyryloxy group, and one benzoyloxy group), there are additional 20 resonances exhibited for the parent skeleton in the ^13^C-NMR spectrum, which comprised four methyls (C-16, C-18, C-19, and C-20), two methylenes (C-1 and C-17), nine methines (C-3, C-4, C-5, C-7, C-8, C-9, C-11, C-12, and C-14), and five quaternary carbons (C-2, C-6, C-10, C-13, and C-15). By comparing the chemical shifts of C-1-C-20 of compound **1** with those of myrsinol diterpenes reported in the literature [4,5,7], the presence of the 10,18-dihydromyrsinol skeleton was obvious. In order to confirm the 10,18-dihydromyrsinol skeleton and determine the positions of the acyloxy groups, the following HMQC and HMBC experiments were performed. By the interpretation of 1D and 2D NMR spectra, the 10,18-dihydromyrsinol skeleton was defined (Figure 1). The HMBC correlations of the carbonyl signal at *δ*_C_ 170.3 with the proton signal at *δ*_H_ 5.48 (H-3) indicated the presence of the one acetoxy group at C-3. Similarly, the long-range couplings of the carbonyl carbon signals at *δ*_C_ 169.2, 170.2, and 165.6 with the proton signals at *δ*_H_ 5.92 (H-5), 4.84 (H-7), and 5.78 (H-14) demonstrated the presence of the two acetoxy groups and one benzoyloxy group at C-5, C-7, and C-14, respectively. The positions of these remaining acyloxy groups were further elucidated by a NOESY experiment. The NOESY correlations of H-14 (*δ*_H_ 5.78) and H-1 *β* (*δ*_H_ 2.44) to the methyl protons (*δ*_H_ 2.09) of the C-15 acetoxy group, and H_3_-19 (*δ*_H_ 1.53) to the methyl protons (*δ*_H_ 2.13) of the C-10 acetoxy group, implied that the two acetoxy groups were located at C-15 and C-10, respectively. Consequently, the isobutyryloxy group could be assigned only to C-2 based on the chemical shift for C-2. By further analyzing the HMQC, HMBC, and ^1^H-^1^H COSY spectra (Figure 2), all the proton and carbon signals were assigned unambiguously. Thus, the planar structure of **1** was established.

**Table 1:** ^1^H-NMR spectroscopic data for compounds **1**–**3** (*δ* ppm in CDCl_3_ and *J* in Hz).

Position		1	2	3
1*α*		3.14 d (17.2)	3.85 d (16.8)	3.57 d (17.6)
*β*		2.44 d (17.2)	2.13 d (16.8)	2.51 d (17.6)
3		5.48 d (3.9)	5.22 br s	5.47 d (3.7)
4		3.68 dd (11.0, 3.9)	3.00 d (11.0)	3.92 d (11.0, 3.70)
5		5.92 d (11.0)	5.83 d (11.0)	6.02 d (11.0)
7		4.84 d (6.5)	4.79 d (7.0)	4.89 d (6.6)
8		6.17 dd (10.2, 6.5)	6.12 dd (9.8, 7.0)	6.18 dd (10.0, 6.6)
9		5.90 dd (10.2, 5.6)	5.90 dd (9.8, 5.6)	5.90 dd (10.0, 5.6)
11		3.17 dd (5.6, 3.0)	3.13 d (5.6)	3.16 d (5.6)
12		3.13 d (3.0)	3.10 s	3.20 s
14		5.78 s	4.05 d (9.0)	5.81 s
16		1.31 s	1.38 s	1.46 s
17		4.09 d (8.8)	3.99 d (8.7)	4.18 d (8.8)
		3.49 d (8.8)	3.47 d (8.7)	3.53 d (8.8)
18		1.62 s	1.58 s	1.55 s
19		1.53 s	1.48 s	1.62 s
20		1.20 s	1.38 s	1.15 s
2-OR	2/6	2.24 q (7.0)	2.41 q (7.1)	7.62 d (7.3)
	3/5	0.83 d (7.0)	1.08 d (7.1)	6.95 t (7.3)
	4	1.08 d (7.0)	1.10 d (7.1)	7.31 t (7.3)
3-OR	2	2.05 s	2.33 q (8.1)	2.09 s
	3		1.11 t (8.1)	
5-OAc	2	1.98 s	1.93 s	2.04 s
7-OAc	2	1.96 s	1.91 s	1.99 s
10-OAc	2	2.13 s	2.04 s	2.17 s
14-OR	2/6	8.09 d (7.2)	2.85 d (9.0) (14-OH)	7.72 d (7.4)
	3/5	7.42 t (7.2)		7.33 t (7.4)
	4	7.56 t (7.2)		7.52 t (7.4)
15-OAc	2	2.09 s	1.91 s	2.09 s

The relative configuration of **1** was elucidated based on the NOESY spectrum and the comparison of NMR data with those of reported myrsinol diterpenes in the literature [4,5,7,13,14]. For all of the reported natural myrsinol diterpenes, the three rings (5/7/6) comprising the myrsinol-skeleton are *trans*-fused with each other, H_3_-16, H-12, the side chain at C-11, and the acyloxy group at C-15 are biogenetically *β*-oriented, and H-4 and H_2_-17 are biogenetically *α-*oriented [7,13]. NOESY correlations observed for H-3/H-4, H-5/H-12, H-12/H-14, H-7/H_2_-17, and H-12/H_3_-20, suggested that H-3, H-4, and H-7 were in *α*-positions, and H-5, H-12, H-14, and H_3_-20 were in *β*-positions. These assignments were consistent with the configuration of reported 10,18-dihydromyrsinol diterpenes [7,13]. Therefore, the structure of compound **1** was characterized as 2*α*-*O*-isobutyryl-3*β*,5*α*,7*β*,10,15*β*-penta-*O*-acetyl-14*α*-*O*-benzoyl-10,18-dihydromyrsinol.

Compound **2** possessed a molecular formula of C_35_H_50_O_14_ based on the HR-ESI-MS (*m*/*z* 717.3101 [M + Na]^+^, calcd. for C_35_H_50_O_14_ Na 717.3098). The ^1^H- and ^13^C-NMR spectra of **2** were very similar to those of compound **1**, which implied that compound **2** should also be a 10,18-dihydromyrsinol diterpene. Close similarities of the chemical shifts from C-1 to C-20 in **2** with those in **1** (Table 2) suggested that compounds **2** and **1** had the same parent skeleton [4,5,7]. In addition to the 20 skeletal carbons, the ^13^C-NMR spectrum revealed additional 15 carbons for the substituent groups, which were deduced and defined as four acetoxy groups, one isobutyryloxy group, and one propinoyloxy group based on the analyses of HMQC and HMBC spectroscopic data of **2** [4,5,7]. The locations of the six acyloxy groups were elucidated by the interpretation of the HMBC spectrum as in the case of **1**. By the HMBC correlations of the protons H-3 (*δ*_H_ 5.22), H-5 (*δ*_H_ 5.83), and H-7 (*δ*_H_ 4.79), with the corresponding carbonyl signals at *δ*_C_ 173.4, 169.1, and 170.2, the two acetoxy groups and the propinoyloxy group were attributed to C-5, C-7, and C-3, respectively. There were no long-range correlations observed for H-14 (*δ*_H_ 4.05) to the carbonyl carbons of the acyloxy groups, which indicated that the substituent at C-14 was a hydroxy group based on the chemical shift for C-14. The remaining two acetoxy groups and one isobutyryl group were assigned to C-10, C-15, and C-2, respectively, which was supported by the NOESY correlation of the H-14 (*δ*_H_ 4.05) and H-1*β* (*δ*_H_ 2.13) to the methyl protons (*δ*_H_ 1.91) of the C-15 acetoxy group and the H_3_-19 (*δ*_H_ 1.48) to the methyl protons (*δ*_H_ 2.04) of the C-10 acetoxy group. The similar myrsinol-type diterpene skeleton of **2** compared to those of compound **1** implied the same *trans*-fusion of the three rings (5/7/6) [7,13]. The NOESY correlations of H-3/H-4, H-5/H-12, H-14/H-12, H-7/H_2_-17, and H-12/H_3_-20 suggested that the C-3 propinoyloxy group and the C-7 acetoxy group were *β*-oriented, while the C-5 acetoxy group and C-14 hydroxy group were *α*-oriented. Thus, compound **2** was elucidated as 2*α*-*O*-isobutyryl-3*β*-*O*-propionyl-5*α*,7*β*,10,15*β*-tetra-*O*-acetyl-10,18-dihydromyrsinol.

Compound **3** was obtained as a white powder. The molecular formula of **3** was determined to be C_44_H_50_O_15_ by HR-ESI-MS (*m*/*z* 841.3040 [M + Na]^+^, calcd. for C_44_H_50_O_15_Na 841.3047), which was compatible with the NMR data. On comparing the chemical shifts for the skeletal carbons in **3** with those for C-1-C-20 in compound **1**, the almost coincidence implied that compounds **3** and **1** had the same characteristic 10,18-dihydromyrsinol skeleton [4,5,7]. The only difference between **3** and **1** was that the one isobutyryloxy group in **1** was replaced by a benzoyloxy group in **3**, which was deduced based on the ^13^C-NMR spectrum and the chemical shifts for the benzoyloxy groups in the reported myrsinol diterpenes [7,13]. Following the same NMR procedures used for **1**, the locations of the acyloxy groups in **3** were determined by the analysis of the HMBC spectrum, which revealed that the five acetoxy groups were attached at C-3, C-5, C-7, C-10, and C-15, and the two benzoyloxy groups were at C-2 and C-14, respectively. The same relative configuration to be inferred for compound **3** and compound **1** was revealed by the careful comparison of the NOESY spectra of **3** and **1**. The structure of compound **3** was therefore characterized as 2*α*,14*α*-di-*O*-benzoyl-3*β*,5*α*,7*β*,10,15*β*-penta-*O*-acetyl-10,18-dihydromyrsinol.

**Table 2:** ^13^C-NMR spectroscopic data for compounds **1**-**3** (*δ* ppm in CDCl_3_).

Position	1	2	3	Position		1	2	3
1	47.3 CH_2_	46.2 CH_2_	46.2 CH_2_	2-OR	1	175.3 C	175.1 C	129.8 C
2	86.3 C	86.8 C	87.4 C		2/6	34.3 CH	34.6 CH	129.3 CH
3	77.7 CH	78.6 CH	78.7 CH		3/5	18.2 CH_3_	18.8 CH_3_	128.0 CH
4	47.6 CH	44.9 CH	47.5 CH		4	18.8 CH_3_	18.9 CH_3_	132.5 CH
5	68.5 CH	68.2 CH	68.5 CH		7			164.6 C
6	53.3 C	53.9 C	53.4 C	3-OR	1	170.3 C	173.4 C	170.5 C
7	62.7 CH	62.9 CH	62.8 CH		2	21.0 CH_3_	27.9 CH_2_	22.3 CH_3_
8	125.7 CH	125.4 CH	125.6 CH		3		8.7 CH_3_	
9	129.8 CH	130.3 CH	129.0 CH	5-OAc	1	169.2 C	169.1 C	169.3 C
10	85.7 C	85.8 C	85.7 C		2	20.9 CH_3_	20.8 CH_3_	20.8 CH_3_
11	44.5 CH	44.0 CH	44.5 CH	7-OAc	1	170.2 C	170.2 C	170.3 C
12	36.9 CH	36.6 CH	36.9 CH		2	20.8 CH_3_	20.7 CH_3_	20.8 CH_3_
13	90.0 C	90.3 C	90.0 C	10-OAc	1	168.4 C	168.9 C	168.3 C
14	73.4 CH	71.2 CH	73.1 CH		2	22.2 CH_3_	22.3 CH_3_	22.2 CH_3_
15	89.9 C	89.7 C	89.8 C	14-OR	1	129.6 C		130.8 C
16	18.8 CH_3_	18.4 CH_3_	18.8 CH_3_		2/6	129.9 CH		129.6 CH
17	69.8 CH_2_	69.6 CH_2_	69.8 CH_2_		3/5	128.3 CH		128.1 CH
18	25.1 CH_3_	24.9 CH_3_	25.1 CH_3_		4	133.2 CH		132.7 CH
19	21.2 CH_3_	21.0 CH_3_	21.0 CH_3_		7	165.6 C		165.7 C
20	24.1 CH_3_	24.9 CH_3_	24.1 CH_3_	15-OAc	1	170.6 C	170.3 C	170.4 C
					2	22.4 CH_3_	22.3 CH_3_	21.1 CH_3_

**Figure 3:**
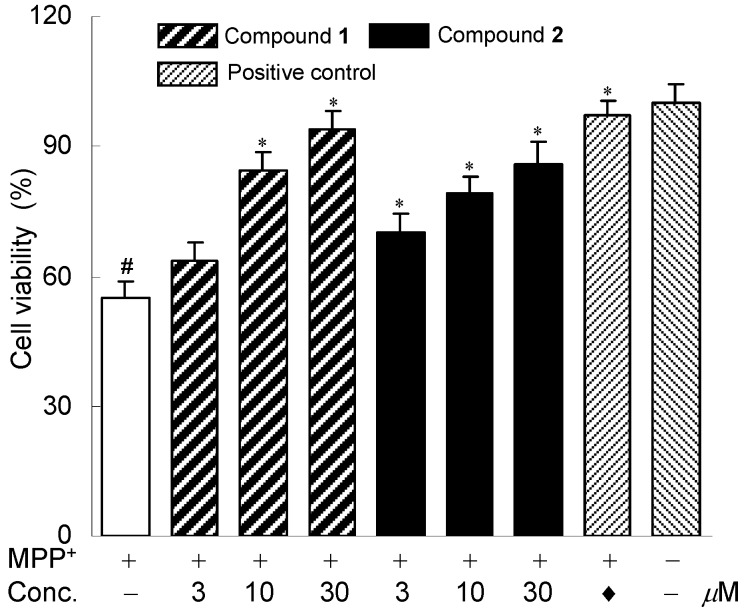
Neuroprotective effects of compounds **1** and **2**. The SH-SY5Y cells were exposed to MPP^+^ and the cell viability was assessed by MTT assay. The cells were treated with 0.8 mM MPP^+^ in the absence or presence of compounds **1** and **2**. Data are expressed as the percentage of values in untreated control cultures. Each value indicates a mean ± SEM (n = 3). ^# ^*p* < 0.05, compared with control group. * *p* < 0.05, compared with the MPP^+^-treated group. ♦ Indicated positive control, bakkenolide H, 15 μM.

In order to explore the potential biological activities of these new diterpenes isolated from the roots of *E. prolifera*, the neuroprotective effects against MPP^+^-induced neuronal cell death in human dopaminergic neuroblastoma SH-SY5Y cells were evaluated as described previously [11,15]. Bakkenolide H was used as a positive control [16]. Compounds **1** and **2** exhibited neuroprotective activities against MPP^+^-induced neuronal cell death in SH-SY5Y cells. The neuroprotective effects of the two compounds were shown in Figure 3. MTT assay indicated that compounds **1** and **2** (3–30 μM) neither affected the cell viability nor showed significant cytotoxicity with the absence of MPP^+^ (data not shown). Compound **3** was not investigated for the neuroprotective effects because of inadequate amount.

## 3. Experimental

### 3.1. General Experimental Procedures

The IR spectra were taken on a on a Bruker Tensor 27 FT-IR spectrometer with KBr discs. Optical rotations were measured in CH_2_Cl_2_ using an Autopol IV automatic polarimeter. The ESI-MS spectra were obtained on a LCQ-Advantage mass spectrometer made by Finnigan Company of America. HR-ESI-MS spectra were obtained using an Ionspec 7.0 T FTICR MS. 1D and 2D NMR spectra were recorded on a Bruker AV 400 instrument (400 MHz for ^1^H and 100 MHz for ^13^C) with TMS as an internal standard. HPLC separations were performed on a CXTH system (Beijing Chuangxintongheng instruments Co. Ltd., China), equipped with a UV3000 detector at 210 nm, and a YMC-pack ODS-AM (250 × 20 mm) column. Silica gel (200–300 mesh, Qingdao Marine Chemical Group Co. Ltd., China) was used for column chromatography. Chemical reagents for isolation were of analytical grade and purchased from Tianjin Yuanli Co. Ltd., China. Biological reagents were from Sigma Company. Human dopaminergic SH-SY5Y cells were obtained from Shanghai Institutes for Biological Sciences, Chinese Academy of Sciences (China). Biological reagents were from Sigma Company.

### 3.2. Plant Material

The roots of *Euphorbia prolifera* Buch-Ham were collected from Kunming, Yunnan Province, China, in July 2010. The botanical identification was made by Dr. Yuanqiang Guo (College of Pharmacy, Nankai University, China), and a voucher specimen (No. 20100705) was deposited at the laboratory of the Research Department of Natural Medicine, College of Pharmacy, Nankai University, China.

### 3.3. Extraction and Isolation

The air-dried roots of *E. prolifera* (3.8 kg) were powdered and extracted with MeOH (3 × 20 L) under reflux. The solvent was evaporated to obtain a crude extract (900 g). The extract was suspended in H_2_O (0.9 L) and partitioned with EtOAc (3 × 0.9 L) to afford the EtOAc soluble part after removing the solvents. The EtOAc soluble part (150.0 g) was subjected to a silica gel column chromatography using a gradient solvent system from 1-40% acetone in petroleum ether to give eight fractions (F_1_-F_8_) based on TLC analyses. Fraction F_8_ was fractionated by MPLC over ODS eluting with a step gradient from 60% to 90% MeOH in H_2_O to obtain four subfractions (F_8-1_-F_8-4_). Subfraction F_8-3_ was purified by preparative HPLC (YMC-pack ODS-AM, 20 × 250 mm, 73% MeOH in H_2_O) to afford compound **1** (16.5 mg). The further purification of F_8-4_ with the same HPLC system using 78% MeOH in H_2_O, resulted in the isolation of compound **3** (3.2 mg). Fraction F_7_, using the same MPLC (60-90% MeOH in H_2_O), provided four subfractions F_7-1-_F_7-4_, and the further purification of F_7-2_ by the above HPLC system (70% MeOH in H_2_O) afforded compound **2** (11.9 mg).

*2α-O-isobutyryl-3β,5α,7β,10,15β-penta-O-acetyl-14α-O-benzoyl-10,18-dihydromyrsinol* (**1**). Colorless needles (MeOH); m.p. 105-107 °C; [α]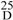 −34.5 (*c* = 0.17, CH_2_Cl_2_); IR (KBr) *ν*_max_ cm^−1^: 2982, 1739, 1602, 1452, 1372, 1246; ESI-MS: *m/z* 807 [M + Na]^+^; HR-ESI-MS *m*/*z* 807.3195 [M + Na]^+^, calcd. for C_41_H_52_O_15_Na 807.3204; ^1^H-NMR (400 MHz, CDCl_3_) data see Table 1, ^13^C-NMR (100 MHz, CDCl_3_) data see Table 2.

*2α-O-isobutyryl-3β-O-propionyl-5α,7β,10,15β-tetra-O-acetyl-10,18-dihydromyrsinol *(**2**). Colorless needles (MeOH); m.p. 91-93 °C; [α]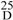 −56.0 (*c* = 0.62, CH_2_Cl_2_); IR (KBr) *ν*_max_ cm^−1^: 3524, 2982, 1735, 1462, 1371, 1244; ESI-MS: *m/z* 717 [M + Na]^+^; HR-ESI-MS *m*/*z* 717.3101 [M + Na]^+^, calcd. for C_35_H_50_O_14_Na 717.3098; ^1^H-NMR (400 MHz, CDCl_3_) data see Table 1, ^13^C-NMR (100 MHz, CDCl_3_) data see Table 2.

*2α,14α-di-O-benzoyl-3β,5α,7β,10,15β-penta-O-acetyl-10,18-dihydromyrsinol* (**3**). White powder; [α]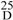 −73.2 (*c* = 0.57, CH_2_Cl_2_); IR (KBr) *ν*_max_ cm^−1^: 2955, 1742, 1452, 1373, 1246; ESI-MS: *m/z* 841 [M + Na]^+^; HR-ESI-MS *m*/*z* 841.3040 [M + Na]^+^, calcd. for C_44_H_50_O_15_Na 841.3047; ^1^H-NMR (400 MHz, CDCl_3_) data see Table 1, ^13^C-NMR (100 MHz, CDCl_3_) data see Table 2.

### 3.4. Bioassay for Neuroprotective Activity

Human dopaminergic neuroblastoma SH-SY5Y cells were cultured at 37 °C in DMEM supplemented with 10% (v/v) inactivated fetal bovine serum and 100 U/mL of penicillin/streptomycin in a water-saturated atmosphere of 95% air and 5% CO_2_. The cells were disassociated by incubation with 1 mM of ethylene glycol-bis(2-aminoethyl ether)-*N*,*N*,*N'*,*N'*-tetraacetic acid (EGTA) in phosphate-buffered saline (PBS) for 15 min and then seeded in 96-well culture plates (1 × 10^4^ cells/well). The cells were incubated at 37 °C in a 5% CO_2_ humidified air incubator for 24 h. The cells were pre-treated for 2 h with various concentrations (3, 10 and 30 μM) of the compounds before incubation in a medium containing 1-methyl-4-phenyl-1,2,3,6-tetrahydropyridine (MPP^+^). MTT dissolved in phosphate-buffered saline was added at the end of incubation to a final concentration of 0.5 mg/mL. After incubation for 4 h at 37 °C and 5% CO_2_, the supernatant was removed and the formed formazan crystals in the viable cells were measured at 490 nm by using a microplate reader (Thermo Fisher Scientific Inc., USA). Experiments were carried out in triplicate. A statistical analysis was performed by a one-way analysis of variance (ANOVA) followed by *post hoc* multiple comparisons using the Student-Newman-Keuls method. Data are expressed as the mean ± SD of three assays. 

## 4. Conclusions

In summary, three new myrsinol diterpenes were successfully isolated from the roots of *E. prolifera*. Their structures were elucidated on the basis of extensive spectroscopic data analyses. Biological evaluation disclosed that two of them exhibited strong neuroprotective effects against MPP^+^-induced neuronal cell death in human dopaminergic neuroblastoma SH-SY5Y cells. The results of our chemical investigation further revealed the chemical constituents of *E. prolifera*. While, the current biological data suggest that myrsinol diterpenes with strong neuroprotective activities may probably be useful for the treatment of nervous system diseases, such as Parkinson's disease [12]. Further biological studies on these compounds are still underway by our group. 

## Supplementary Materials

Supplementary data (1D and 2D NMR spectra and HR-ESIMS spectra) associated with this article can be accessed at: http://www.mdpi.com/1420-3049/17/8/9520/s1.
