# Supplementary material for: Three New Myrsinol Diterpenes from Euphorbia prolifera and Their Neuroprotective Activities
Source: Molecules. 2012 Aug 9;17(8):9520–8. doi: 10.3390/molecules17089520 (PMC6268895; doi:10.3390/molecules17089520)

Contents

S1 $^{1}$H-NMR spectrum for compound **1**
S2 $^{13}$C-NMR spectrum for compound **1**
S3 HMQC spectrum for compound **1**
S4 HMBC spectrum for compound **1**
S5 $^{1}$H-$^{1}$H COSY spectrum for compound **1**
S6 NOESY spectrum for compound **1**
S7 HR-ESI-MS spectrum for compound **1**
S8 $^{1}$H-NMR spectrum for compound **2**
S9 $^{13}$C-NMR spectrum for compound **2**
S10 HMQC spectrum for compound **2**
S11 HMBC spectrum for compound **2**
S12 $^{1}$H-$^{1}$H COSY spectrum for compound **2**
S13 NOESY spectrum for compound **2**
S14 HR-ESI-MS spectrum for compound **2**
S15 $^{1}$H NMR spectrum for compound **3**
S16 $^{13}$C NMR spectrum for compound **3**
S17 HMQC spectrum for compound **3**
S18 HMBC spectrum for compound **3**
S19 NOESY spectrum for compound **3**
S20 HR-ESIMS spectrum for compound **3**

S1 $^{1}$H-NMR spectrum for compound **1**

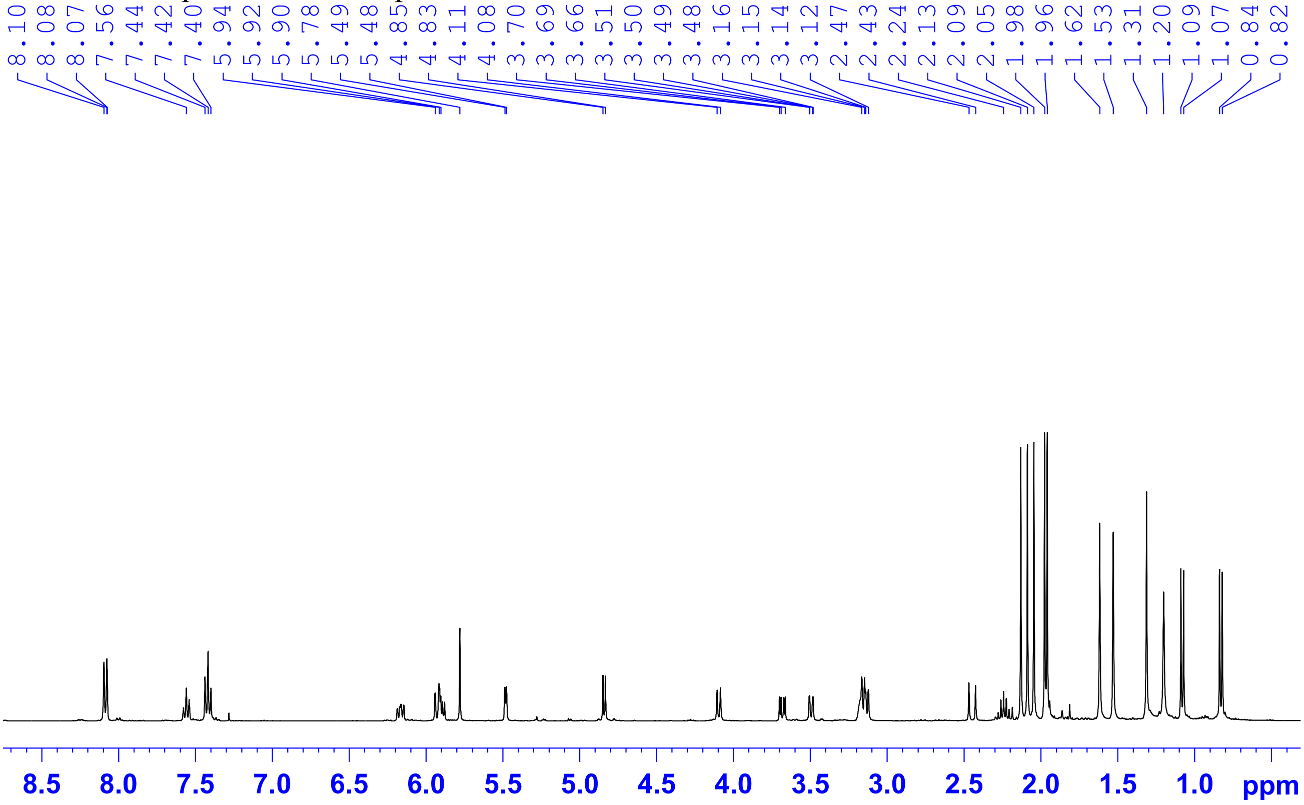


S2 $^{13}$C-NMR spectrum for compound **1**

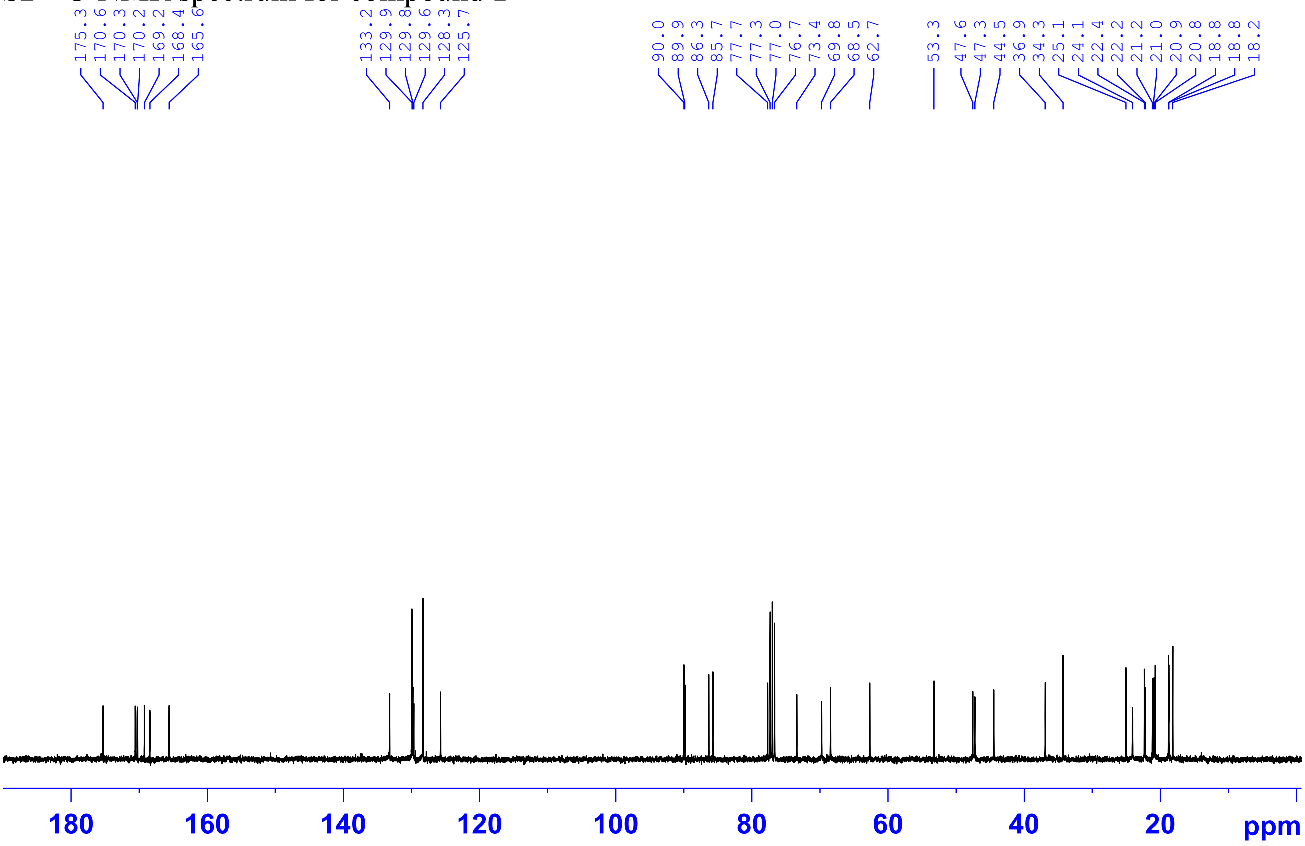

S3 HMQC spectrum for compound **1**

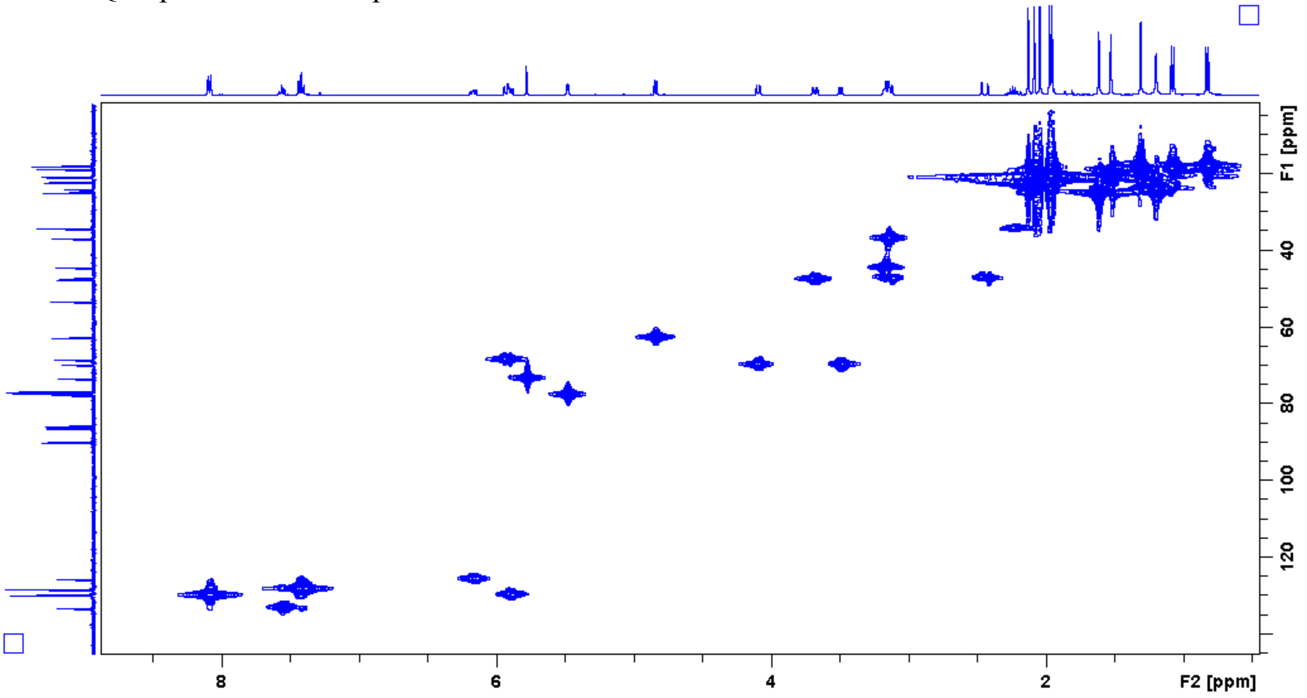


S4 HMBC spectrum for compound **1**

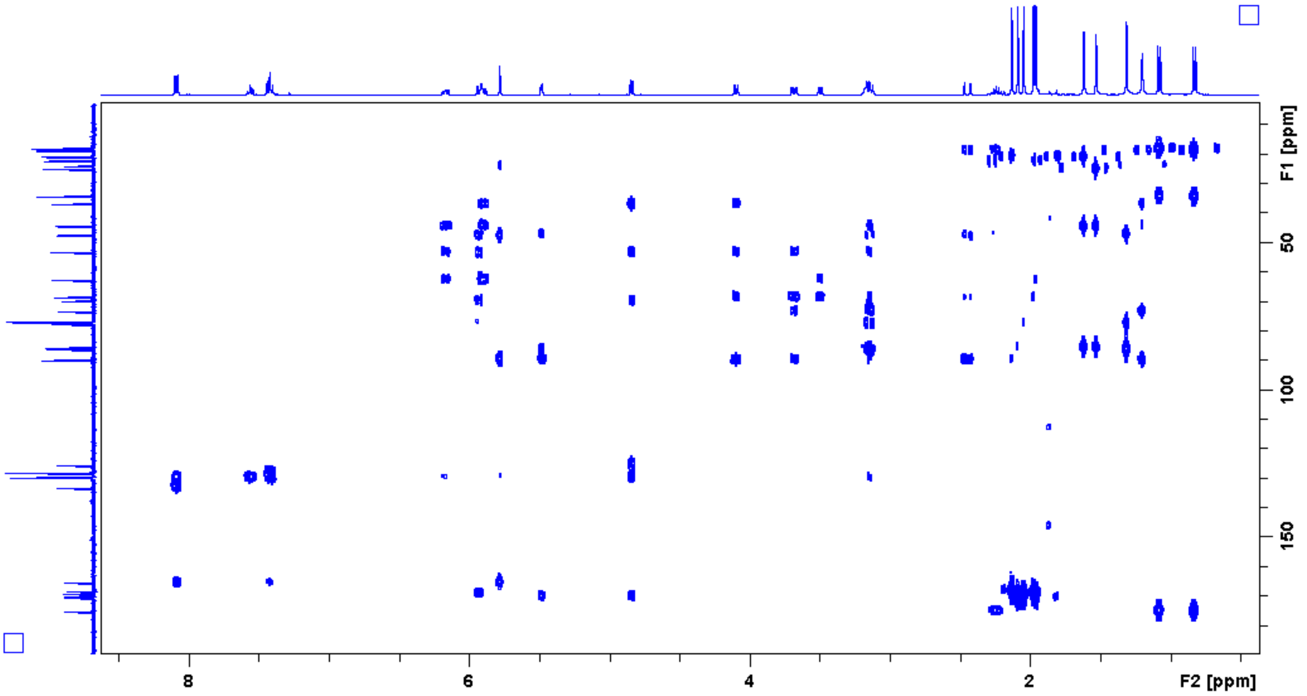

S5 $^{1}$H-$^{1}$H COSY spectrum for compound **1**

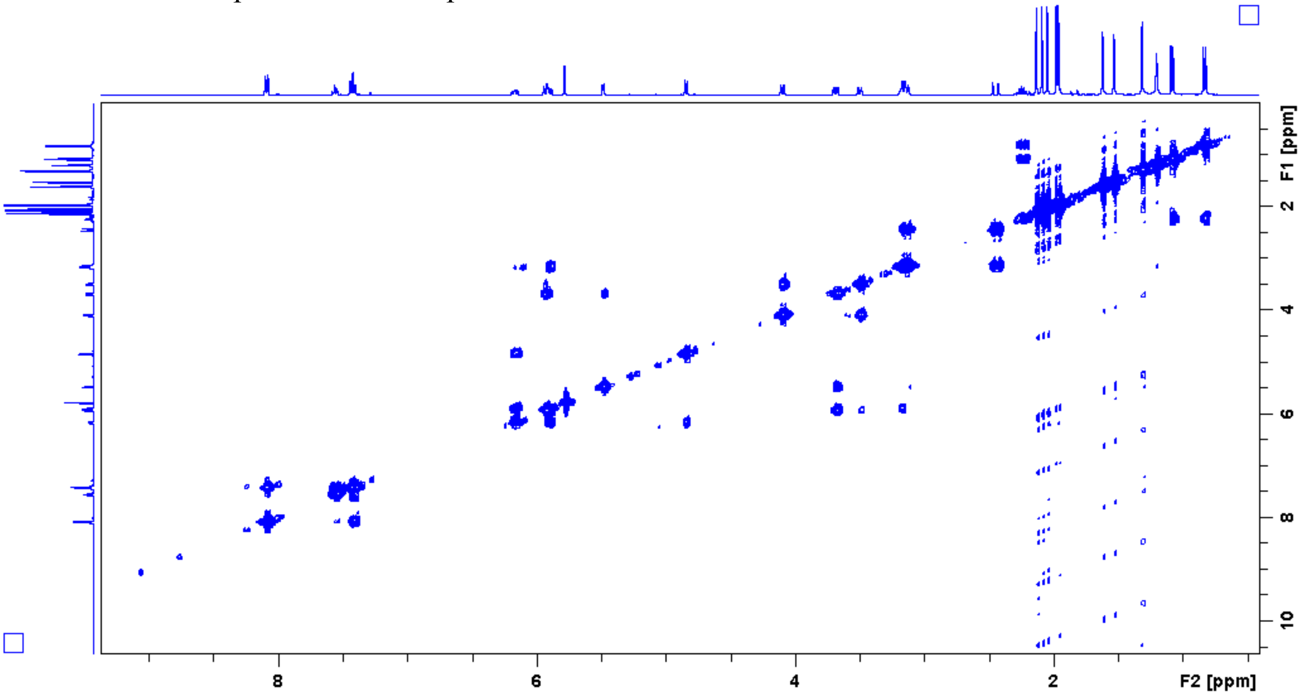


S6 NOESY spectrum for compound **1**

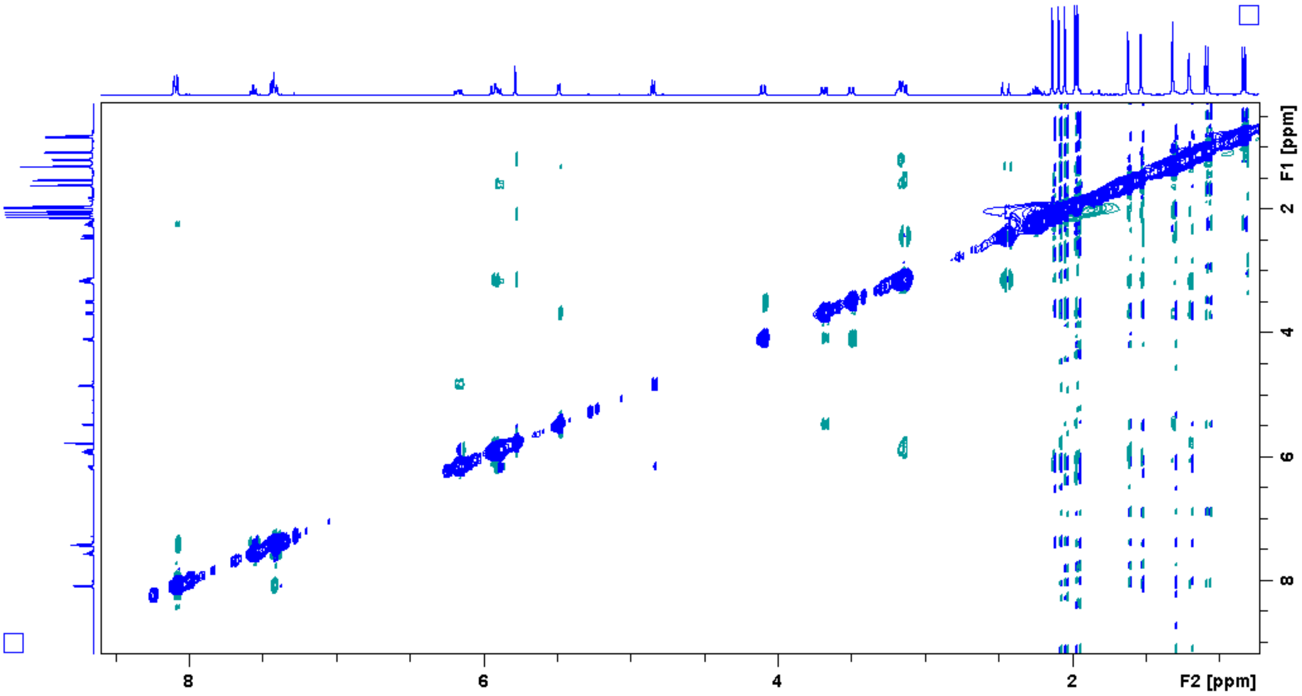

S7 HR-ESI-MS spectrum for compound **1**

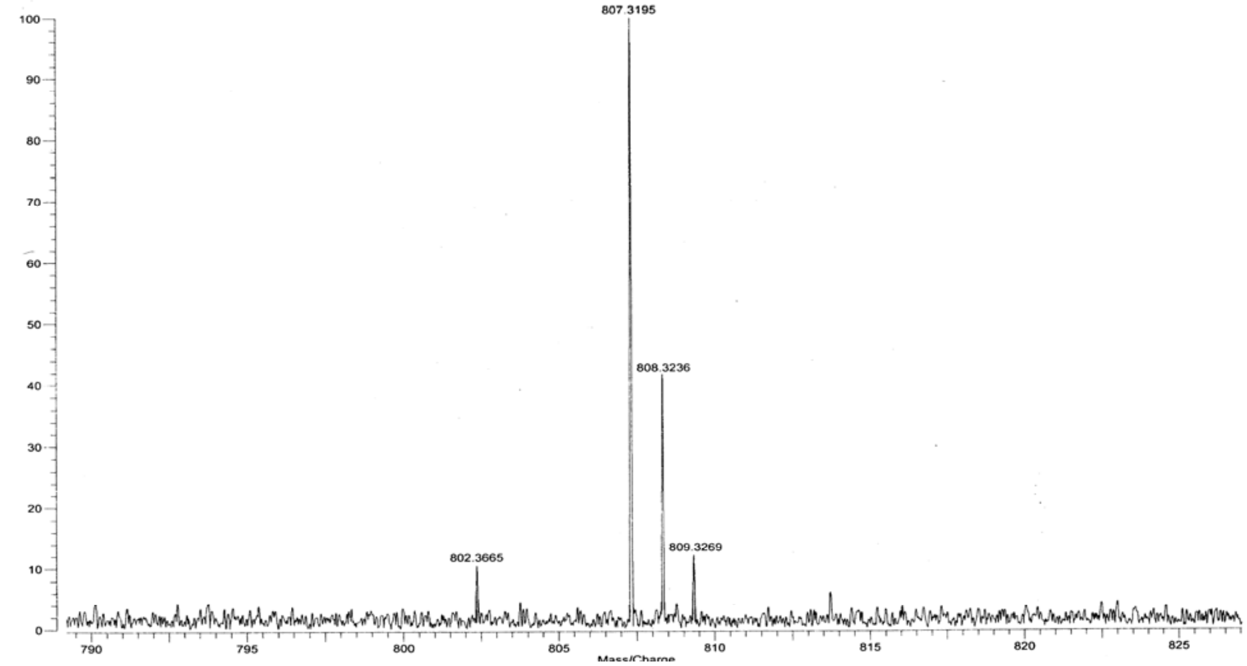


S8 $^{1}$H-NMR spectrum for compound **2**

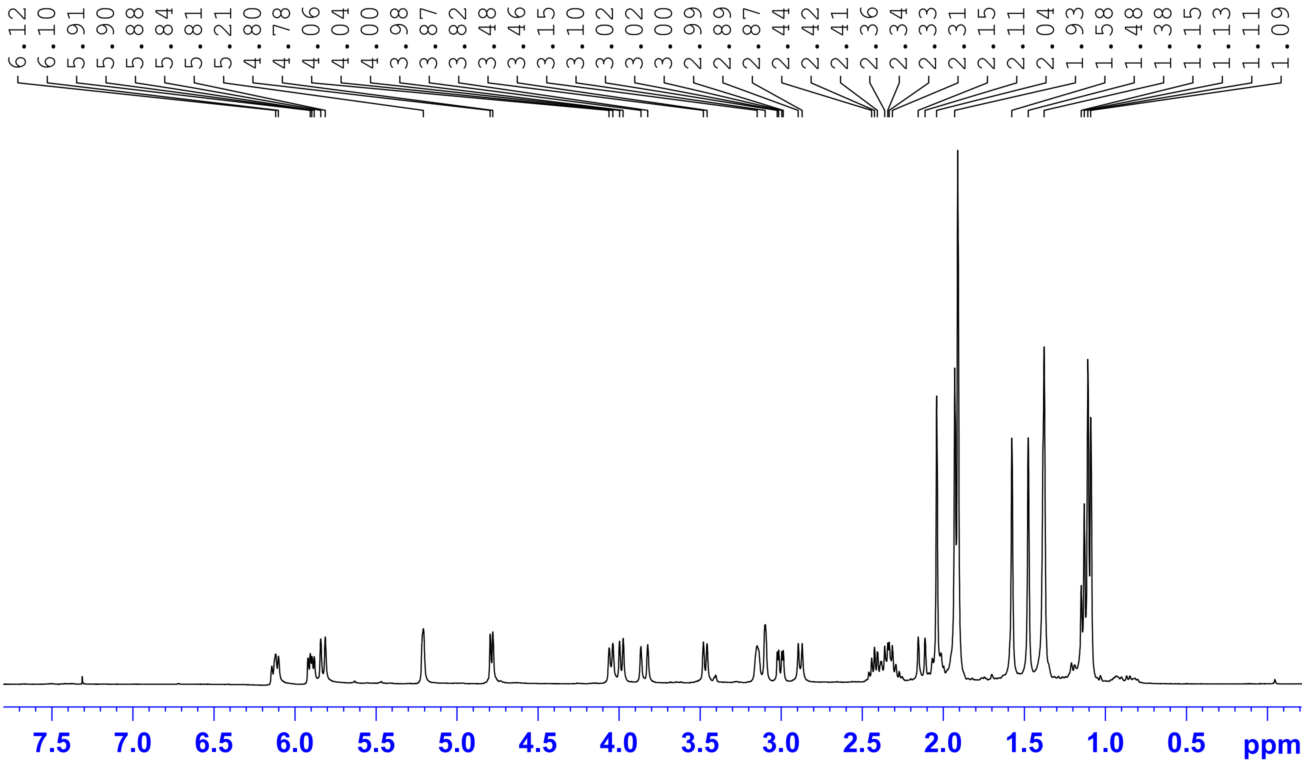

S9 $^{13}$C-NMR spectrum for compound **2**

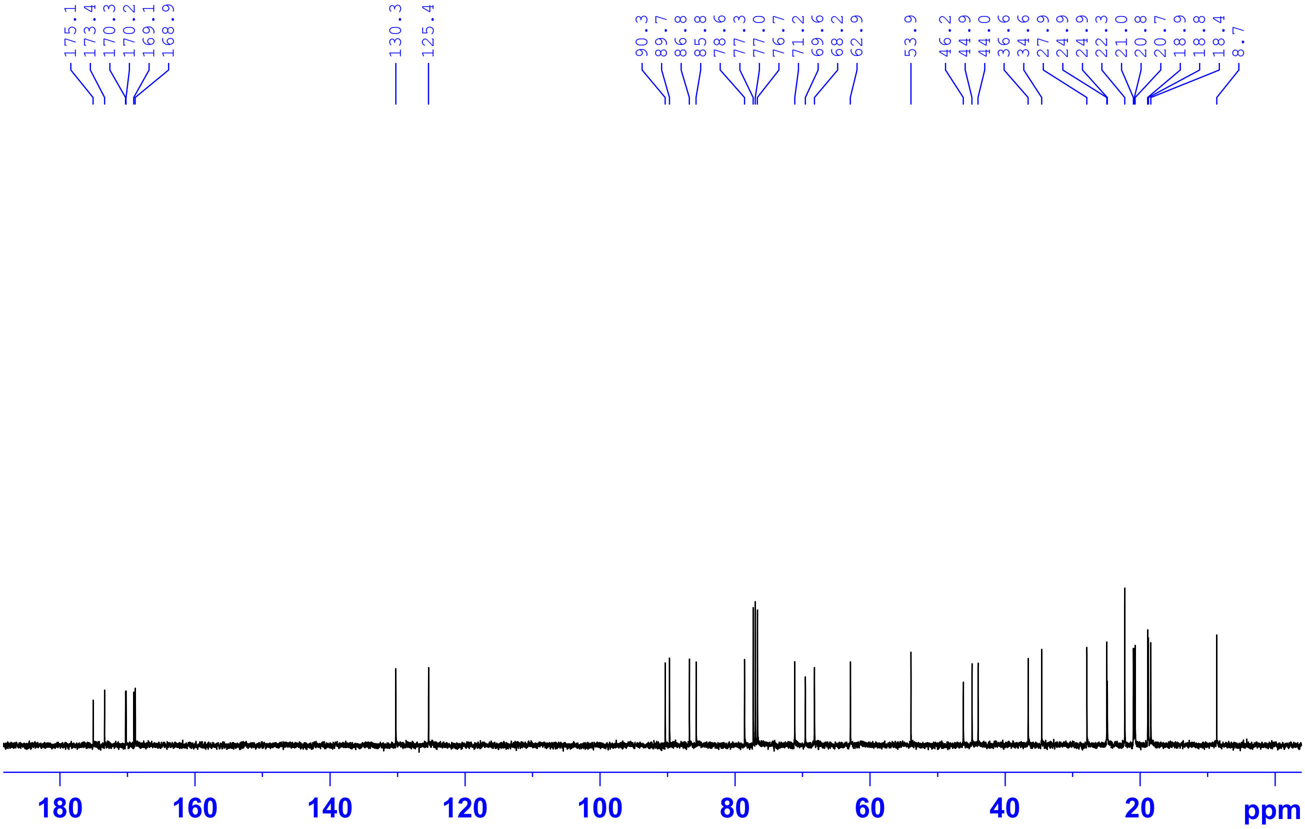


S10 HMQC spectrum for compound **2**

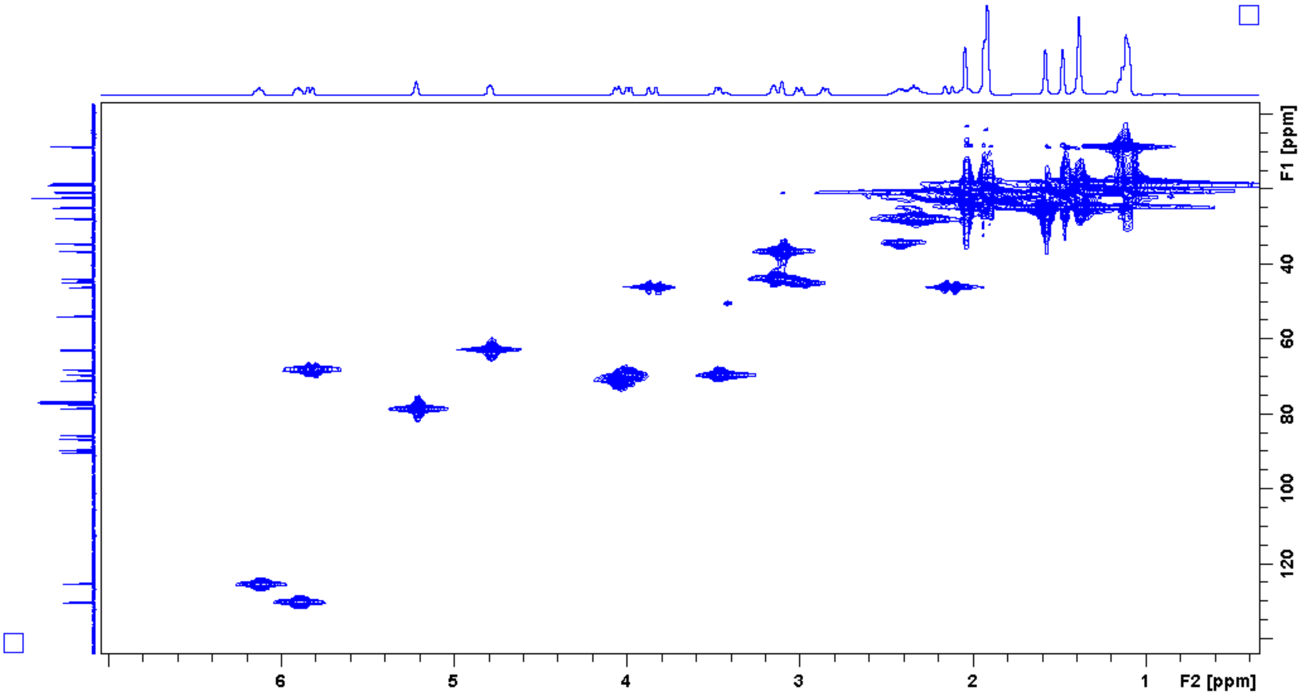

S11 HMBC spectrum for compound **2**

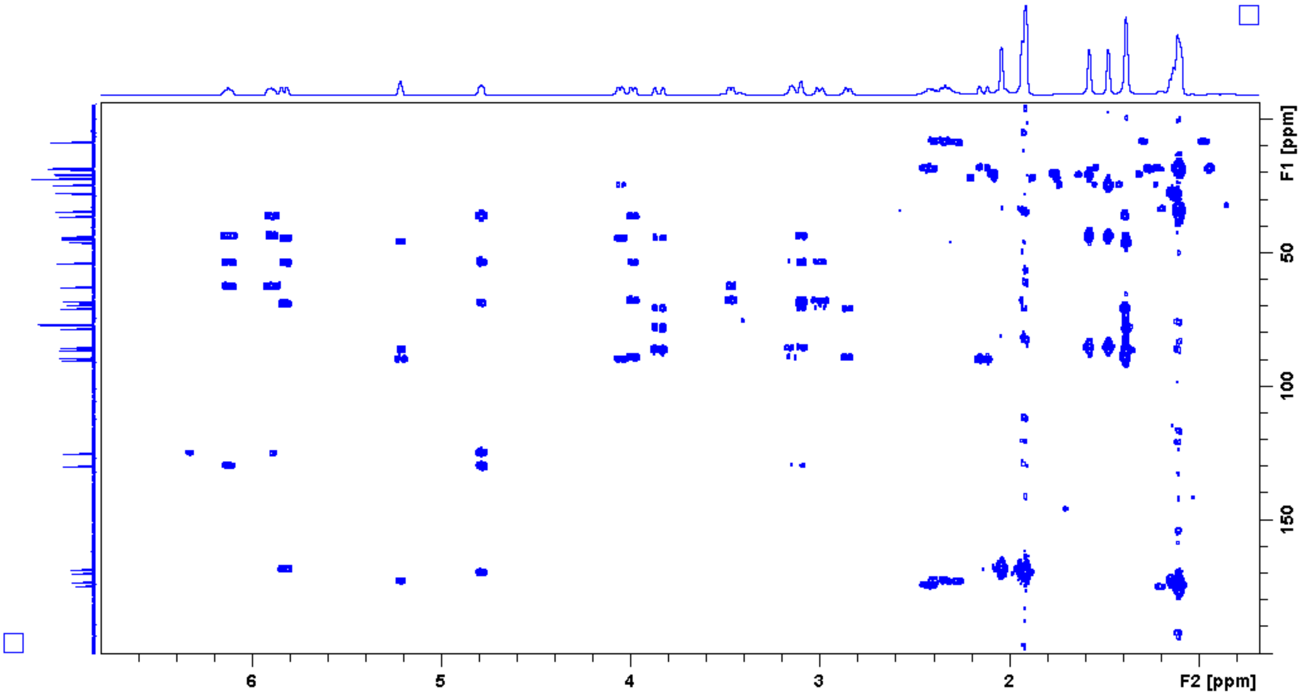


S12 $^{1}H$-$^{1}H$ COSY spectrum for compound **2**

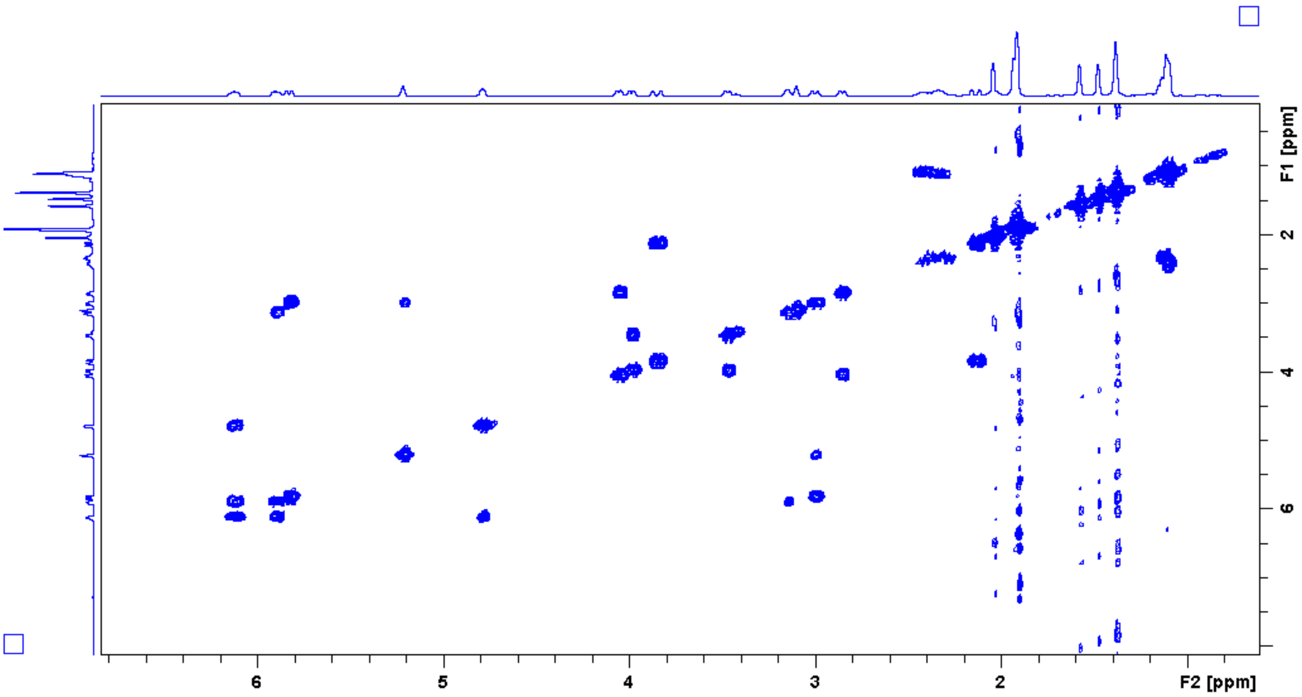

S13 NOESY spectrum for compound **2**

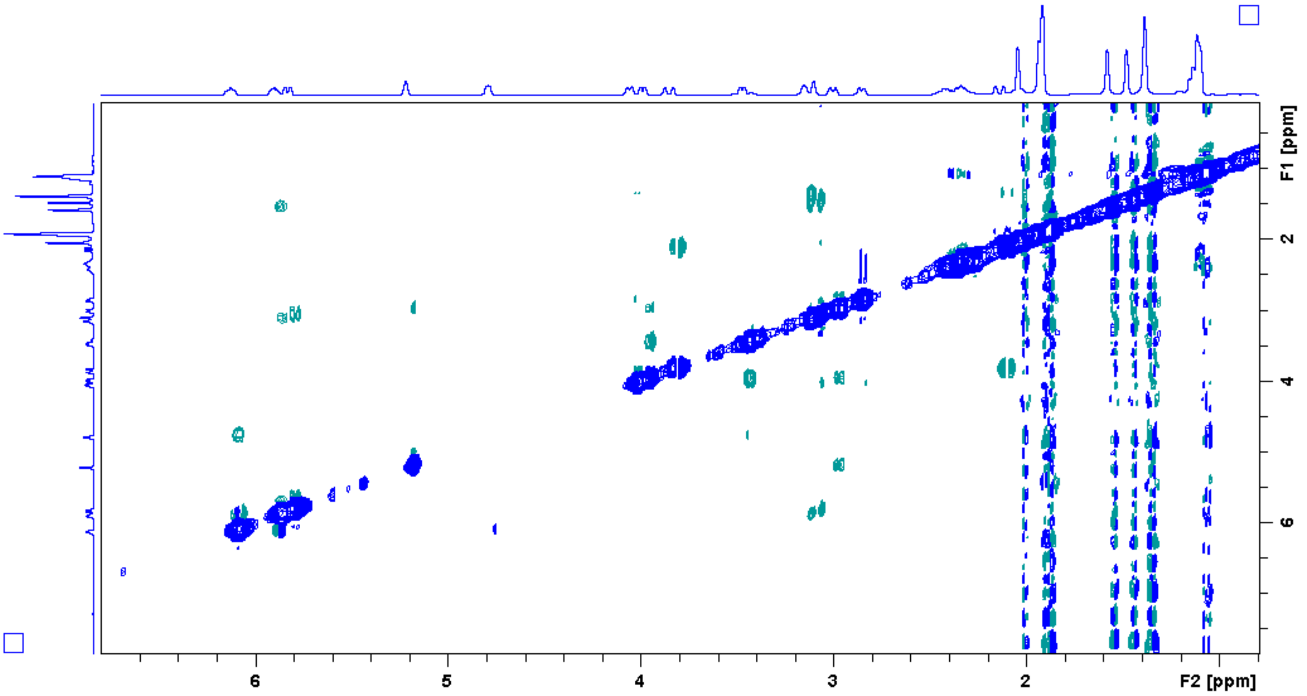


S14 HR-ESI-MS spectrum for compound **2**

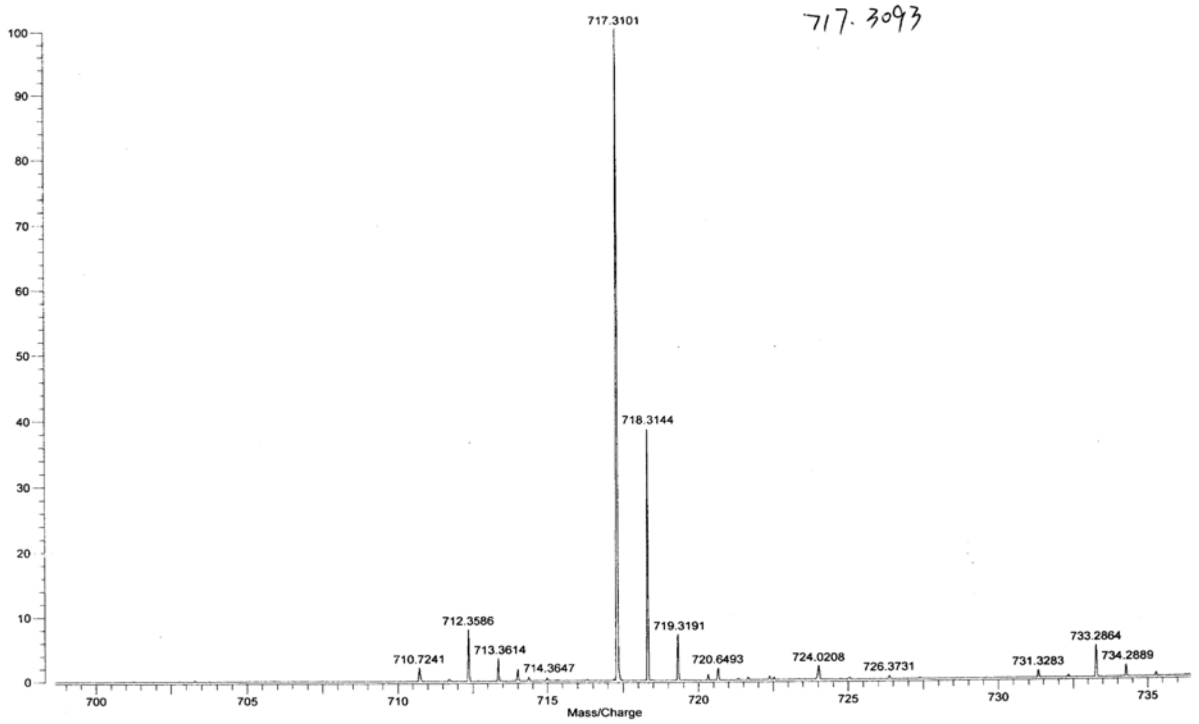

S15 $^{1}$H NMR spectrum for compound **3**

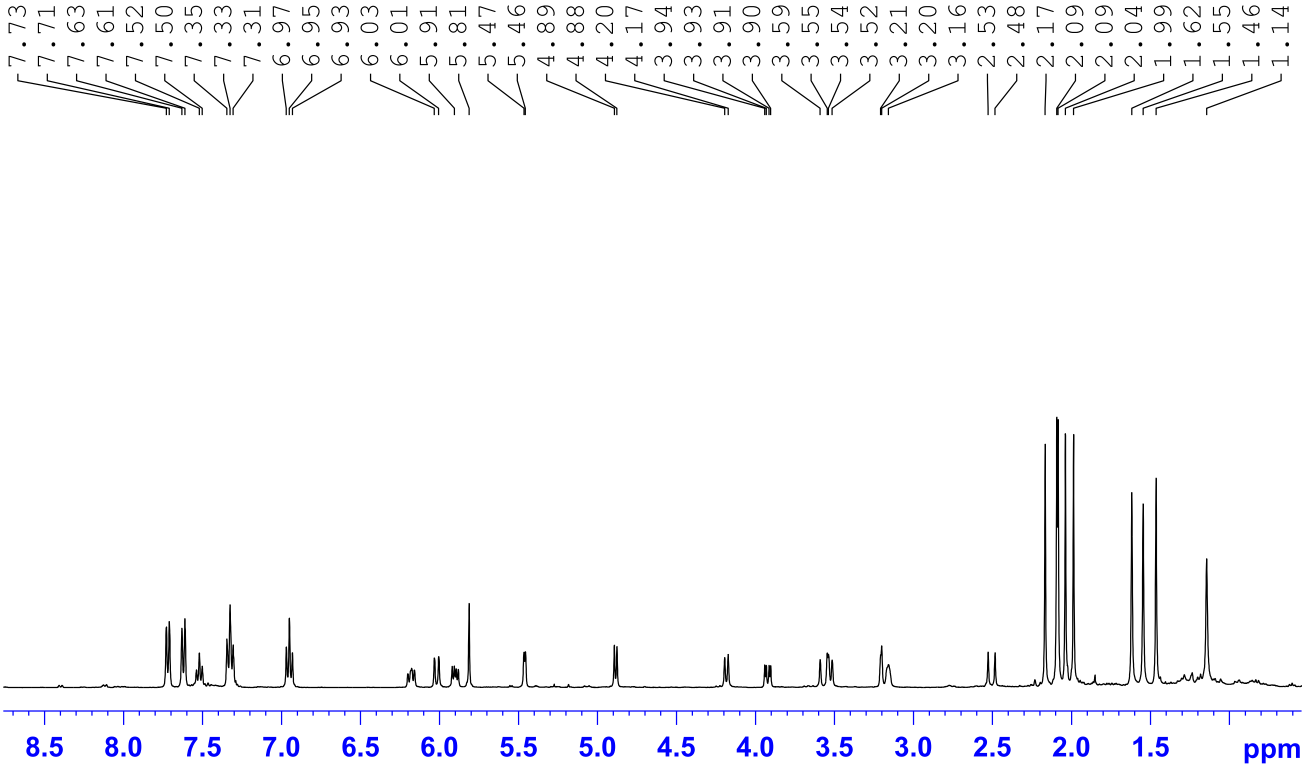


S16 $^{13}$C NMR spectrum for compound **3**

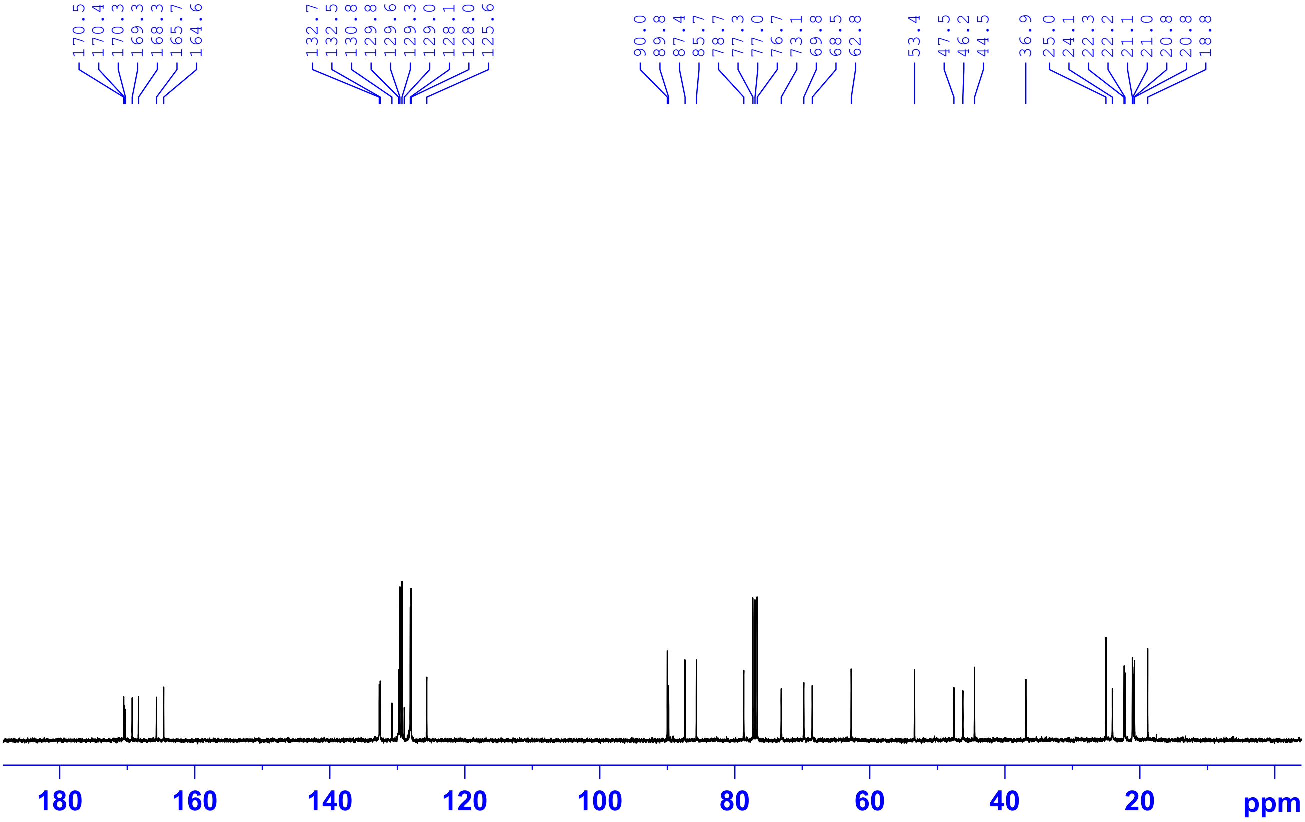

S17 HMQC spectrum for compound **3**

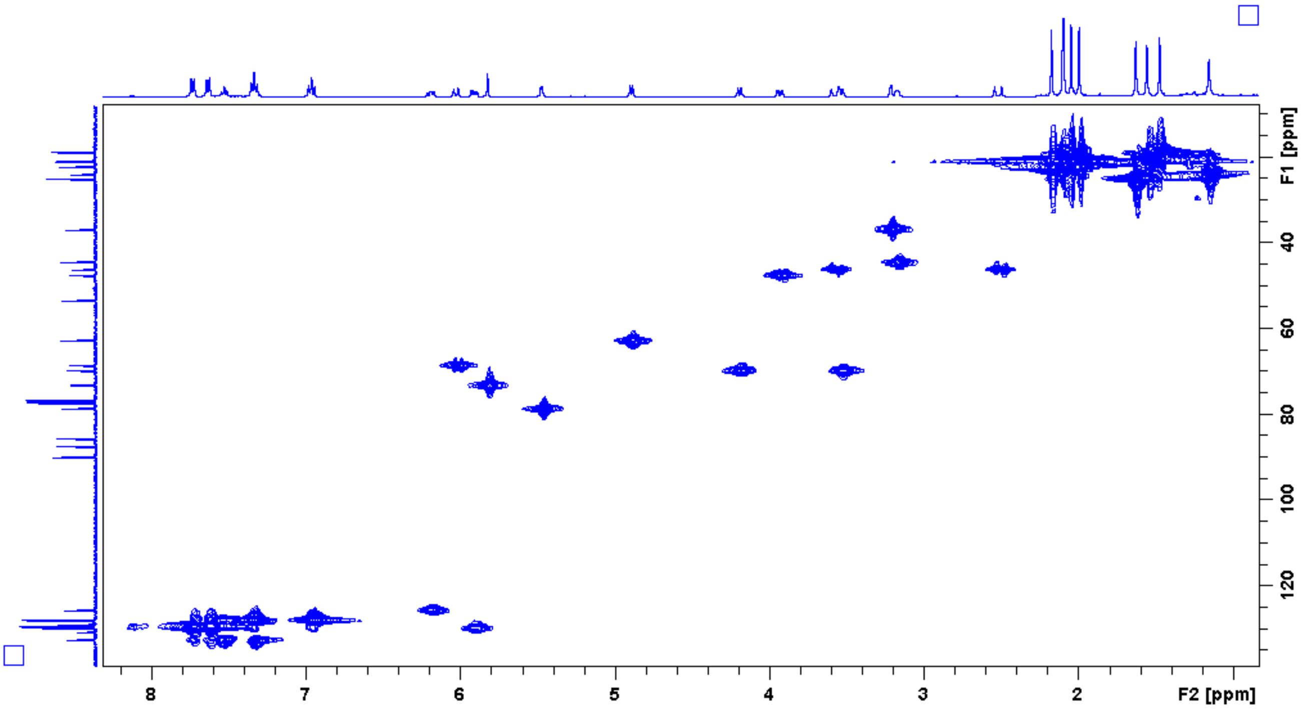


S18 HMBC spectrum for compound **3**

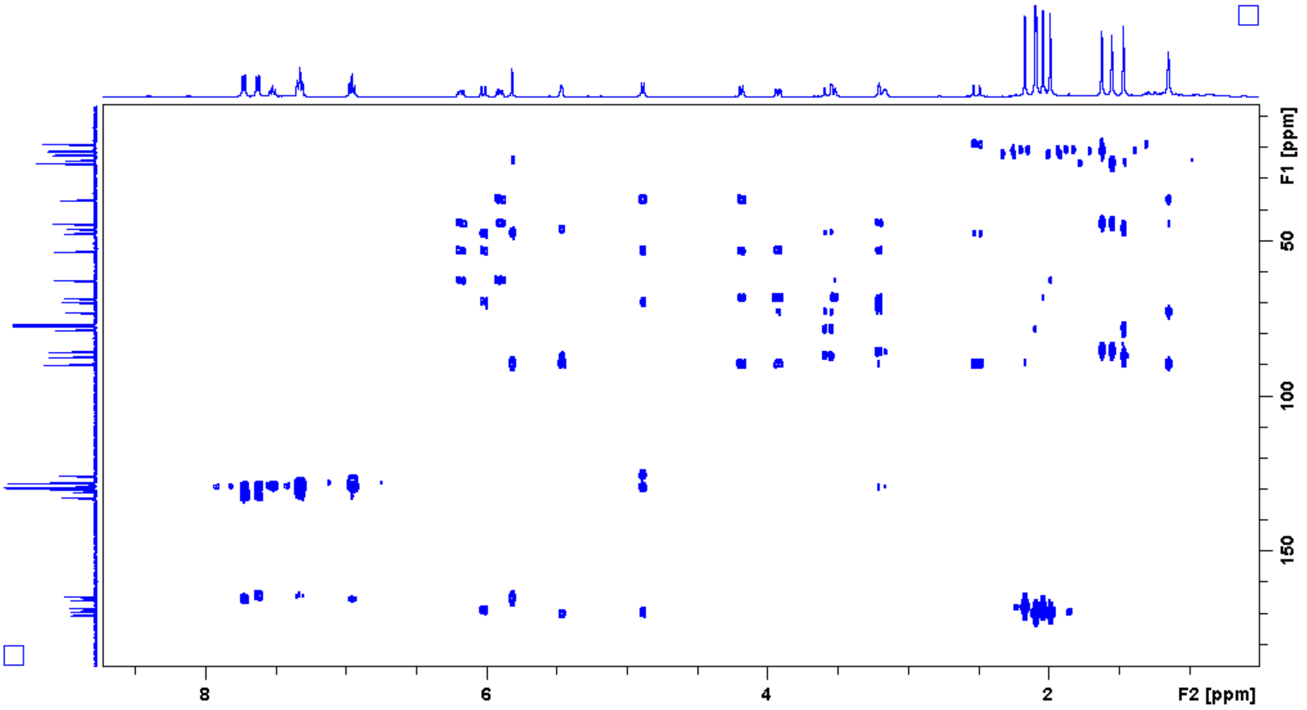

S19 NOESY spectrum for compound **3**

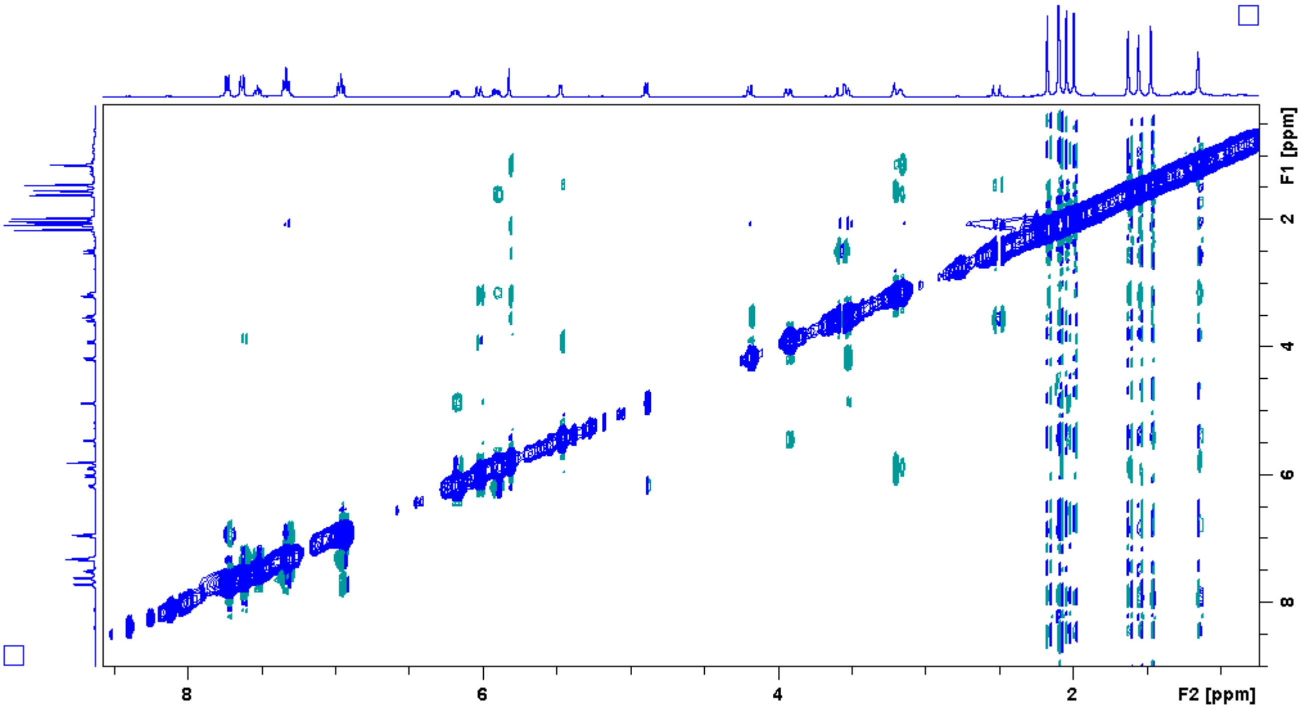


S20 HR-ESIMS spectrum for compound **3**

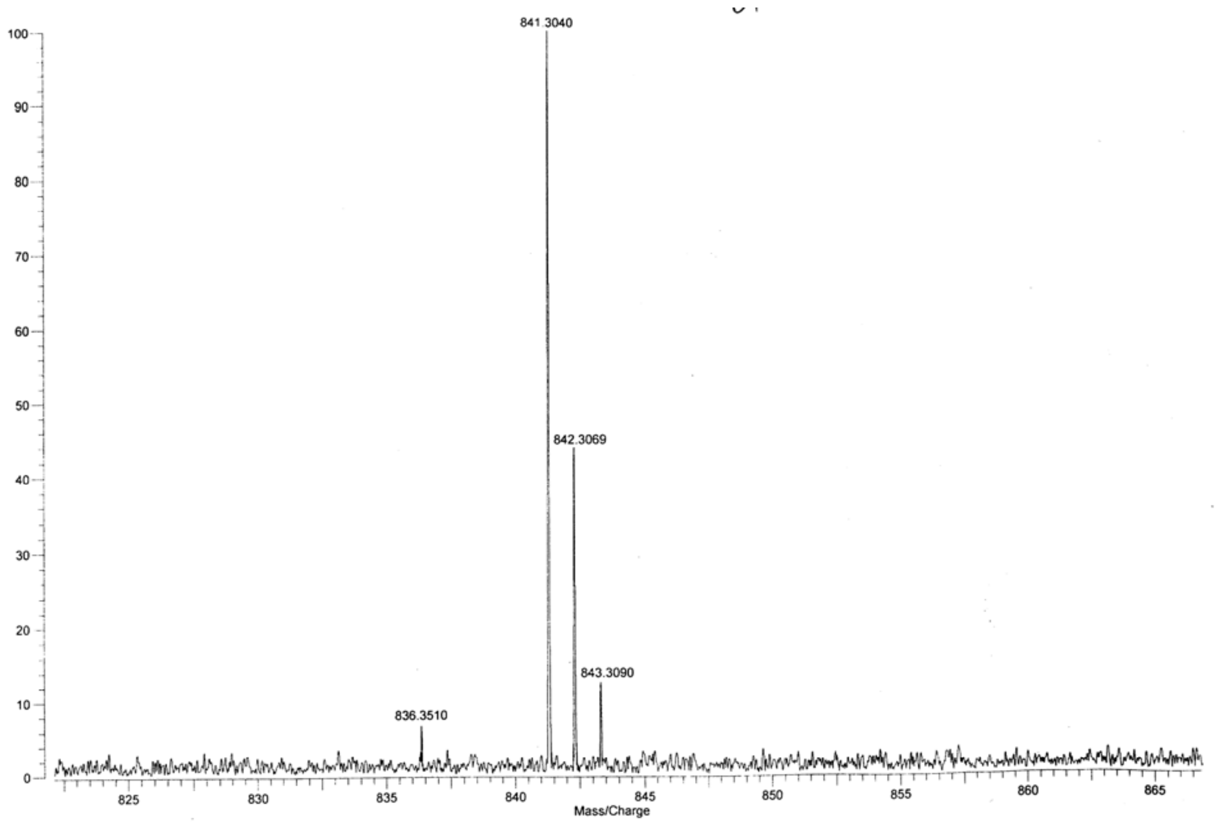

Supplement: Supplementary file 1 [file molecules-17-09520-s001.pdf]
